# Exploiting coherence for real-time studies by single-bunch imaging

**DOI:** 10.1107/S1600577514005852

**Published:** 2014-05-09

**Authors:** A. Rack, M. Scheel, L. Hardy, C. Curfs, A. Bonnin, H. Reichert

**Affiliations:** a European Synchrotron Radiation Facility, 38000 Grenoble Cedex, France

**Keywords:** ultra-fast phenomena, picosecond, X-ray phase contrast, radioscopy, crack propagation, coalescence, fracture

## Abstract

First real-time studies of ultra-fast processes by single-bunch imaging at the European Synchrotron Radiation Facility are reported. By operating the storage ring of the ESRF in single-bunch mode with its correspondingly increased electron bunch charge density per singlet, the polychromatic photon flux density at insertion-device beamlines is sufficient to capture hard X-ray images exploiting the light from a single bunch (the corresponding bunch length is 140 ps FWHM). Hard X-ray imaging with absorption contrast as well as phase contrast in combination with large propagation distances is demonstrated using spatial samplings of 11 µm and 35 µm pixel size. The images acquired allow one to track crack propagation in a bursting piece of glass, breaking of an electrical fuse as well as cell wall rupture in an aqueous foam. Future developments and their potential in the frame of the proposed Phase II of the ESRF Upgrade Program are discussed.

## Abstract 2

First real-time studies of ultra-fast processes by means of a single-bunch imaging technique at the European Synchrotron Radiation Facility are reported. Both absorption contrast and phase contrast are accessible thanks to propagation distances of several metres.

## 1. Introduction   

The ultimate time-resolution for X-ray imaging at a synchrotron light source is given by the bunch structure of the electrons in the storage ring. Hence, exploiting the light emitted by an isolated singlet allows one to acquire snapshot images of temporal events lasting as short as a few tens of picoseconds. Pioneering work carried out at the Advanced Photon Source (Argonne National Laboratory, USA) impressively demonstrated the potential of so-called single-bunch imaging, *e.g.* to study fuel injection (Wang *et al.*, 2008 ▶). In this communication, first real-time studies carried out at the European Synchrotron Radiation Facility (ESRF) by means of a single-bunch imaging technique are presented. A key achievement is the fact that the use of propagation-based phase contrast with large sample–detector distances can be exploited to beat photon flux limitations (Cloetens *et al.*, 1996 ▶; Rack *et al.*, 2010 ▶), *i.e.* phase contrast can relax the demands on photon flux density compared with experiments reported in the literature (Luo *et al.*, 2012 ▶). Single-bunch imaging can therefore be applied on a wider range of beamlines including the possibility of using larger beams with cross sections of up to several square centimeters. Furthermore, the proposed Phase II of the ESRF Upgrade Program will enable other techniques such as spectroscopy and diffraction to be used in single-bunch mode.

For the present experiments, two complementary beamlines were chosen: (i) the High Energy Diffraction and Scattering beamline, ID15A, characterized by its outstanding intense photon flux density and hard X-ray spectrum related to the use of an in-vacuum undulator (Di Michiel *et al.*, 2005 ▶); (ii) the 150 m-long Parallel and Coherent Beam Microtomography beamline, ID19, with its superior coherence properties in combination with a large beam that can reach cross sections of up to several square centimeters. In comparison, beamline ID19 supplies lower photon flux density with respect to ID15A due to the length of the beamline and the available insertion devices (Weitkamp *et al.*, 2010 ▶). In both cases, a polychromatic beam was utilized, *i.e.* the white spectrum emitted by the source filtered by absorbers only, leading to a homogeneous wavefront and maximized photon flux density. Indirect X-ray imaging detectors with a radiation-resistant layout were used for the experiments in combination with fast CMOS-based cameras (Koch, 1994 ▶).

## 2. Experiments   

For the experiments, the ESRF was operated in a dedicated single-bunch machine mode. In the early days of the ESRF, this mode was routinely delivered with storage-ring currents up to 20 mA. Due to the large number of low-aperture chambers installed since then, the coupling impedance of the storage-ring vacuum chamber has increased so much that the current is presently limited to 10–12 mA. The natural purity level of 10^−5^ (*i.e.* the ratio of the number of parasitic electrons in the adjacent bunches to the number of electrons in the main bunch) was considered sufficient for the experiment. However, a ratio of 10^−10^ is routinely achieved for the users and can be produced for further tests. With 10 mA in the single bunch, the measured bunch length is 140 ps FWHM [frequently measured *via* the emitted photons using a streak camera (*cf.* Scheidt, 1996 ▶)] while the energy spread is in the range between 0.0015 (10 mA) and 0.0012 (7 mA). The revolution frequency of the single bunch is determined by the storage-ring circumference (844 m): 355.450 kHz (2.81 µs). During the experiment, the radio frequency (RF) was carefully monitored to be in the range 352.2022831–352.2023137 MHz with an accuracy of 0.1 Hz.

The measurements at ID15A were carried out with white synchrotron radiation. The indirect X-ray image detector operates with a 1× magnification (tandem design of two Kineoptics apo­chromats 75 mm, f/2 aperture, 0.2 numerical aperture). As scintillating material, commercially available bulk LuAG:Ce (500 µm-thick; Crytur, Czech Republic) was chosen, which is known to be suited to fast synchrotron-based imaging involving high heat loads (Di Michiel *et al.*, 2005 ▶). It also features a fast response time that is compatible with single-bunch imaging (Luo *et al.*, 2012 ▶). The white beam of the ID15A insertion device (U22, 6.01 mm gap) was filtered only by the mandatory beamline elements such as windows (1.4 mm diamond and 4.0 mm beryllium), the Laue monochromator (1.0 mm Si) of ID15B, and a fixed absorber (3.5 mm SiC). The resulting flux of the 50 keV beam absorbed by the scintillator at 65 m from the source was 5 × 10^13^ photons s^−1^ mm^−2^ (10 mA ring current). Up to 5 m propagation distance is accessible for phase-contrast imaging. As a high-speed camera, a pco.dimax was used [PCO AG, Germany; 2016 × 2016 pixels, 11 µm pixel size, 50% peak quantum efficiency at 500 nm, 36 GB on-board memory for fast intermediate storage, 1279 full images per second (fps) maximum frame rate]. A region of interest (ROI) of 384 × 200 pixels (horizontal × vertical) was set resulting in a field of view of 4.2 mm × 2.2 mm. Using this ROI the camera can operate at more than 36000 fps, which allows one to catch the bunch every tenth turn. The bunch position was used as a trigger to exploit the maximum intensity. The exposure time was set to 1.28 µs, *i.e.* short enough for imaging only at the bunch pass with a repetition time of 2.817 µs. In the case of continuous illumination, a motion faster than 8 m s^−1^ in the object plane would cause motion blur.

For the single-bunch imaging at ID19 the beamline was operated with up to three insertion devices in series at minimal gap (two U32 undulators with 11.5 mm gap and the W150 wiggler with 26 mm gap) (Weitkamp *et al.*, 2010 ▶). A 1.4 mm Al filter and the mandatory diamond window (1.4 mm-thick) were inserted to suppress the soft part of the emitted spectrum, *i.e.* to avoid unwanted heat load onto the detector. For this polychromatic configuration the energy spread exploitable for imaging is large, ranging roughly from 20 keV to 50 keV with a mean energy of approximately 30 keV. The detector consisted of a long-working-distance objective (OptiquePeter, France; 300 mm focal length, f/5 aperture, 0.1 numerical aperture, nominal 3.5× demagnification) that projects the luminescence image of a 500 µm-thick LuAG:Ce single-crystal scintillator *via* a mirror onto the chip of a pco.dimax camera (same model as for the ID15A experiments). Hence, the detector operated with an effective pixel size of approximately 35 µm. In this configuration, approximately 3 × 10^12^ photons mm^−2^ s^−1^ are stopped by the scintillator screen (6 mA ring current, all three insertion devices in use at minimal gap), *i.e.* the photon flux density is orders of magnitude lower compared with other beamlines applying single-bunch imaging (Luo *et al.*, 2012 ▶). In order to reach the desired imaging frame rate, a ROI [185 × 146 pixels (horizontal × vertical)] needed to be set, *i.e.* the field of view was approximately 6.4 mm × 5.1 mm. No bunch triggering option is currently available at ID19: the pco.dimax acquired continuously images with 2.26 µs exposure time at a frame rate of 35504 images s^−1^ (one ‘single-bunch’ image was acquired every 28 µs). A sample–detector distance of approximately 9 m was used in order to obtain a sufficient fringe-dominated phase contrast in the images for the given pixel size and mean photon energy.

## 3. Results and discussions   

**Figure 1:**
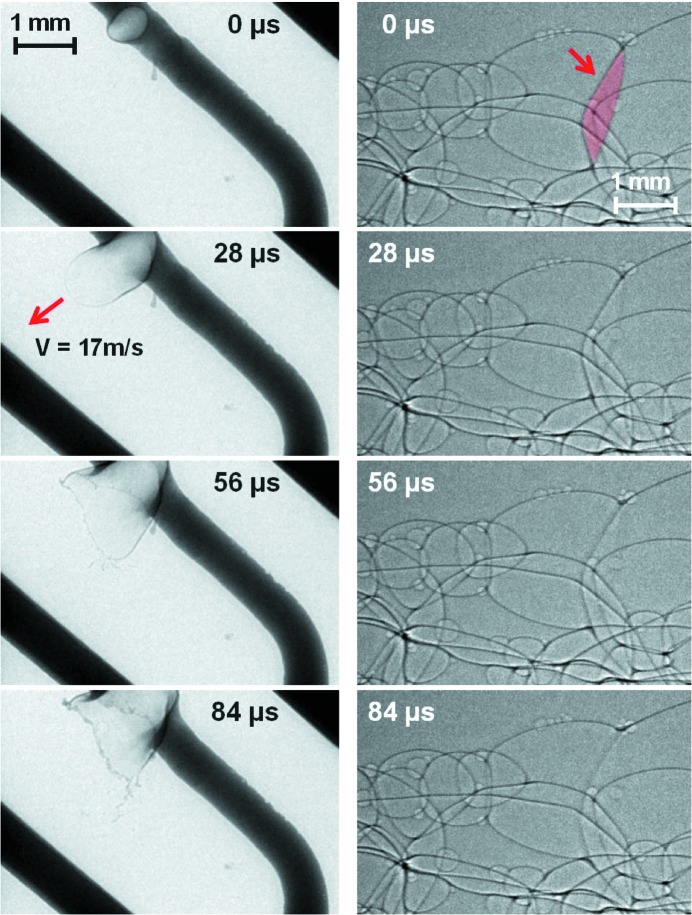
Time series showing pore formation in an electric wire leading to breaking of a fuse (left) and a coalescence event in an aqueous foam (right): the lamella shared by two neighboring pores (red, marked by the arrow) disappears in successive stages from top to bottom. The pictures are single-bunch images acquired at beamline ID15A (ESRF).

Experiments involving fast-moving objects were conducted in order to prove that only one passing bunch with an extraordinary short bunch length of 140 ps contributes to the image formation. For the purpose of recording images dominated by absorption contrast at 50 keV (ID15A) a highly absorbing 10 A car fuse was chosen as the first sample. Through the fuse a 20 A current was applied which caused the fuse to break. In Fig. 1 ▶ (left), images are depicted showing an expanding pore inside the molten metal wire: it is rupturing the wire connection of the fuse (see supplementary movie #1^1^). The images are of remarkably high quality in terms of sharpness and contrast. The arrow labels a metal pore lamella which is expanding at 17 m s^−1^ while still the images show no blurring. There is no ‘double image’ visible. This confirms that the recorded light originates from one electron bunch only, considering that even the short exposure time alone would result in a blurred image at this velocity. After imaging a highly absorbing sample, an aqueous foam with negligible absorption was placed 5 m upstream from the detector. This sample–detector distance introduces significant phase contrast at 50 keV allowing one to image thin aqueous lamellas even at this energy. The images in Fig. 1 ▶ (right) from the recorded video show the coalescence of two adjacent bubbles, *i.e.* the successive disappearance of the edge-enhanced border of a lamella (see supplementary movie #2). Surprisingly, the bursting part is broadening. Since it is known that only light from a single bunch is utilized for image formation, one can exclude motion blur. Hence, the blur must be the redistribution of liquid during the coalescence event. The ID15A results demonstrate that single-bunch imaging is feasible with existing beamlines when relaxing the demands on the spatial resolution with respect to the already published results (Luo *et al.*, 2012 ▶). Higher photon flux density is required to increase the spatial resolution.

**Figure 2:**
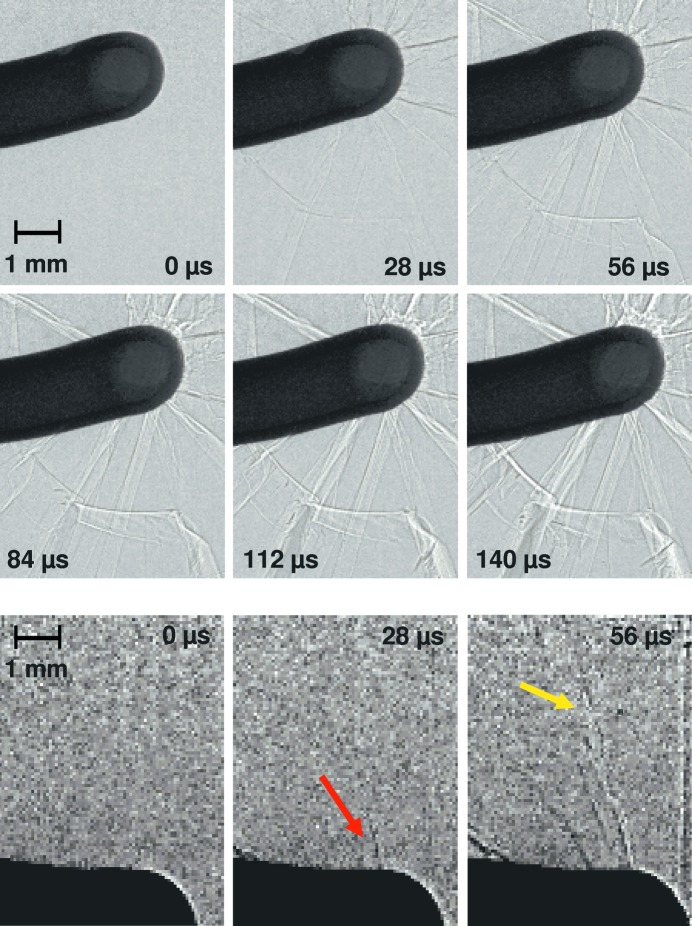
Top: time series recording crack propagation in a glass plate initiated by an accelerated bolt (all three ID19 insertion devices in use). Bottom: time series showing the growth of an individual crack (marked by the arrows: red shows the crack tip, yellow the propagated crack with the tip outside the field-of-view, one ID19 undulator in use). The grey-level contrast has been adapted for better visibility of the crack with respect to the images above. The pictures are acquired *via* single-bunch imaging at beamline ID19 (ESRF).

Consequently, beamline ID19 is considered next featuring a reduced photon flux density but superb coherence properties. In Fig. 2 ▶, time series of images are depicted showing crack propagation in a glass plate, as acquired at ID19. As in the case of the ID15A results, each image represents a snapshot corresponding to light emitted by a singlet in the storage ring passing once through the ID19 insertion devices during the acquisition. The temporal distance between the images (28 µs) is given by the image frame rate of the camera. The glass plate was orientated at an angle of 45° with respect to the incoming X-ray beam (the bolt initiating the cracks translated perpendicularly to the glass plate; the full series of pictures allows us to estimate its velocity to be approximately 11.7 m s^−1^) (see supplementary movie #3). In the case of continuous illumination, the highest velocity enabling sharp images to be recorded is given by the pixel size (having taken into account the 45° inclination angle) and the exposure time, *i.e.* 35 µm × 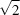/2.26 µs 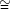 21.9 m s^−1^. Considering cracks propagate at the speed of sound (∼4000 m s^−1^ for glass), *i.e.* orders of magnitude faster, while the images acquired are still ‘frozen in time’, the outstanding temporal resolution due to the exploitation of the electron’s bunch structure in the storage ring is clearly demonstrated. Our results confirm that the combination of single-bunch exposure, long propagation distances and large-pixel-size detectors is ideally matched to acquire images with an exploitable contrast. Hence, it is now possible to reach an ultimate time solution with a synchrotron light source at beamline ID19 for a broad range of materials imaging challenges. Higher (coherent) photon flux densities especially at shorter wavelengths are required now to study denser materials and/or to increase the spatial resolution. Studies such as the one presented here can be employed, for example, to understand energy dissipation in solid matter, an important process that, for instance, drives dust cloud creation during a volcanic eruption (Dürig & Zimanowski, 2012 ▶), or to understand crack propagation in semiconductor materials (Danilewsky *et al.*, 2013 ▶).

**Figure 3:**
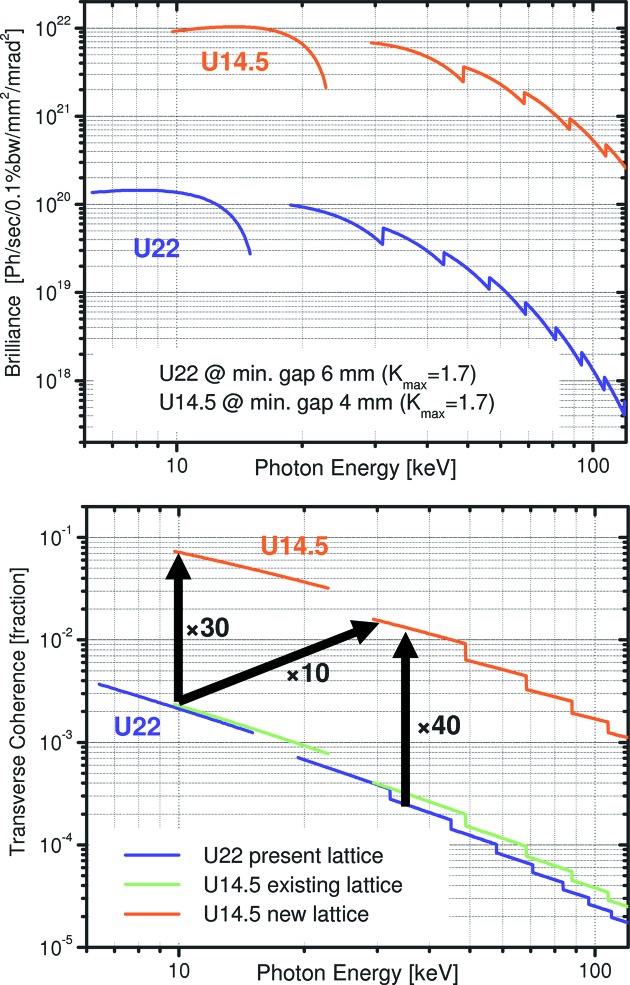
Comparison of the brilliance (top) and coherent fraction of photons (bottom) between a conventional U22 ESRF undulator (existing lattice) and the recently developed cryogenic permanent-magnet undulator U14.5 (anticipated new lattice).

Today’s X-ray imaging experiments with ultimate time resolution on storage rings are mainly limited by the available source brilliance and detector performance in terms of both spatial and time resolution. Currently, a worldwide race has started towards the construction of diffraction-limited storage-ring-based light sources featuring advanced multi-bend achromat lattice designs. This new generation of synchrotron radiation sources will offer an increase of about two orders of magnitude in source brilliance that translates directly into a corresponding increase of the coherent fraction of X-ray beams. The simultaneous increase of brilliance and coherence will greatly facilitate ultrafast imaging applications down to the (sub)-100 ps time scale. The first example of these next-generation sources is the proposed construction of a novel storage ring in the existing ring tunnel of the ESRF. The target horizontal emittance is of the order of 100 pm, down by a factor of 40 from today’s 4 nm. The corresponding increase in coherence is linear in this range and leads directly to a contrast enhancement. Fig. 3 ▶ shows the X-ray source parameters for the next generation of small-gap short-period insertion devices at the ESRF in comparison with an existing device in the current storage ring. The enhanced performance is immediately apparent and should trigger the evolution of ultrafast imaging from a niche application toward a routine tool in materials science, even at photon energies substantially above those reported in this communication. Another exciting opportunity for future materials science applications is the realistic prospect of single-bunch diffraction. First attempts at the Advanced Photon Source (Luo *et al.*, 2012 ▶) and the ESRF (unpublished) have actually delivered scattering patterns, though the intensity level still needs to be improved for real materials science applications. The expected performance of new near-diffraction-limited light sources constitutes thus a big step towards new applications of single-bunch diffraction, spectroscopy and imaging for materials science.

## Footnotes

1 Supporting information for this paper is available from the IUCr electronic archives (Reference: MO5082).
